# COP Cells in States of Bone Anabolism and Abnormal Calciﬁcation/Ossification

**DOI:** 10.1093/geroni/igab046.169

**Published:** 2021-12-17

**Authors:** Robert Pignolo

**Affiliations:** Mayo Clinic College of Medicine, Rochester, Minnesota, United States

## Abstract

Circulating osteogenic progenitor (COP) cells are a population of cells in the peripheral blood with the capacity for bone formation, as well as broader differentiation into mesoderm-like cells in vitro. There are several pathologies of accelerated bone formation and physiological responses to injury in which COP cells have been theorized to play a role. These include fracture, vascular calciﬁcation, and subtypes of heterotopic ossiﬁcation (HO). Overall, the available studies suggest COP cells are likely to be mobilized in response to fracture, home to the site of injury, undergo a maturation process, and contribute to the osteogenesis and angiogenesis required for fracture healing. HO is the pathological process of bone formation in nonskeletal tissue and can be acquired or hereditary. COP cells may seed sites of injury and inﬂammation that precede the formation of endochondral bone identiﬁed in both genetic and nongenetic forms of HO. Vascular calciﬁcation is a common occurrence in older adults and is strongly associated with poorer cardiovascular health outcomes. It appears that COP cells, particularly those expressing hematopoietic and vascular markers such as CD45 and CD34, contribute to the calciﬁcation and ossification of atherosclerotic plaques and aortic valves, and that they correlate to the severity of the calciﬁcation. Whether COP cells are attracted to sites of injury and inﬂammation and so are highly associated with fracture, vascular calcification/ossification and HO, or whether they underlie these processes at a more mechanistic level, remains to be more clearly demonstrated.

